# The incidence of non-affective psychotic disorders in Chile between 2005 and 2018: results from a national register of over 30 000 cases

**DOI:** 10.1017/S0033291720002664

**Published:** 2022-04

**Authors:** Alfonso González-Valderrama, Hannah E. Jongsma, Cristián Mena, Carmen Paz Castañeda, Rubén Nachar, Juan Undurraga, Nicolás Crossley, David Aceituno, Barbara Iruretagoyena, Carlos Gallardo, Pilar Mondaca, Matías Monje, Matías Irarrazaval, Cynthia Zavala, Lucia Valmaggia, James B. Kirkbride

**Affiliations:** 1 Early Intervention Program, Instituto Psiquiátrico Dr J Horwitz Barak, Santiago, Chile; 2 School of Medicine, Universidad Finis Terrae, Santiago, Chile; 3 Psylife Group, Division of Psychiatry, University College London, London, UK; 4 Department of Neurology and Psychiatry, Faculty of Medicine, Clínica Alemana Universidad del Desarrollo, Santiago, Chile; 5 Department of Psychiatry, School of Medicine, Pontificia Universidad Católica de Chile, Santiago Metropolitan Region, Chile; 6 Biomedical Imaging Center, Pontificia Universidad Católica de Chile, Santiago Metropolitan Region, Chile; 7 Department of Psychosis Studies, King's College London, Institute of Psychology, Psychiatry, and Neuroscience, London, UK; 8 Department of Health Service and Population Research, King's College London, Institute of Psychology, Psychiatry, and Neuroscience, London, UK; 9 Department of Mental Health, Ministry of Health, Santiago, Chile; 10 Millennium Institute for Research in Depression and Personality, Santiago, Chile; 11 Faculty of Medicine, Clínica Psiquiátrica Universitaria, University of Chile, Santiago, Chile; 12 Department of Psychology, King's College London, Institute of Psychology, Psychiatry, and Neuroscience, London, UK

**Keywords:** Case register, Chile, incidence, non-affective psychosis, South America

## Abstract

**Background:**

Evidence suggests the incidence of non-affective psychotic disorders (NAPDs) varies across persons and places, but data from the Global South is scarce. We aimed to estimate the treated incidence of NAPD in Chile, and variance by person, place and time.

**Methods:**

We used national register data from Chile including all people, 10–65 years, with the first episode of NAPD (*International Classification of Diseases, Tenth Revision:* F20–F29) between 1 January 2005 and 29 August 2018. Denominators were estimated from Chilean National Census data. Our main outcome was treated incidence of NAPD and age group, sex, calendar year and regional-level population density, multidimensional poverty and latitude were exposures of interest.

**Results:**

We identified 32 358 NAPD cases [12 136 (39.5%) women; median age-at-first-contact: 24 years (interquartile range 18–39 years)] during 171.1 million person-years [crude incidence: 18.9 per 100 000 person-years; 95% confidence interval (CI) 18.7–19.1]. Multilevel Poisson regression identified a strong age–sex interaction in incidence, with rates peaking in men (57.6 per 100 000 person-years; 95% CI 56.0–59.2) and women (29.5 per 100 000 person-years; 95% CI 28.4–30.7) between 15 and 19 years old. Rates also decreased (non-linearly) over time for women, but not men. We observed a non-linear association with multidimensional poverty and latitude, with the highest rates in the poorest regions and those immediately south of Santiago; no association with regional population density was observed.

**Conclusion:**

Our findings inform the aetiology of NAPDs, replicating typical associations with age, sex and multidimensional poverty in a Global South context. The absence of association with population density suggests this risk may be context-dependent.

## Introduction

The most recent international meta-analysis on the incidence of psychotic disorders demonstrated substantial variation in rates in studies conducted since 2002 (Jongsma, Turner, Kirkbride, & Jones, 2019). Although methodological differences in study design and case finding may account for some of this variation (Jongsma et al., 2019), it also provides an entry point for enquiry about population-level differences which may be relevant to the aetiology of psychotic disorders. For example, a recent multinational comparison of the incidence of psychotic disorders across 17 settings in six countries demonstrated sevenfold variation in rates using a standardized methodology (Jongsma et al., 2018). Further analyses of these data suggested that this variation correlated with area-level socioeconomic affluence (Jongsma et al., 2018) and background levels of cannabis use in the population (Di Forti et al., 2019).

In particular, non-affective psychotic disorders (NAPDs) have shown more spatial variation than their affective counterparts (March et al., 2008), and many studies – predominantly conducted in the Global North – have suggested that this is associated with population density (Mortensen et al., 1999), deprivation (Hakulinen, Webb, Pedersen, Agerbo, & Mok, 2019; Richardson, Hameed, Perez, Jones, & Kirkbride, 2018), social fragmentation (Allardyce et al., 2005), residential mobility (Price, Dalman, Zammit, & Kirkbride, 2018) and inequality (Burns, Tomita, & Kapadia, 2014). Nonetheless, robust evidence from countries outside of the Global North is scarce. For example, in the aforementioned international meta-analysis (Jongsma et al., 2019), only 24 of 177 (13.5%) incidence studies conducted between 2002 and 2017 were conducted outside of Europe, North America and Australia. Data from these studies suggested that incidence rates of all psychotic disorders were not demonstrably higher or lower in low- and middle-income countries (LMICs) compared with high-income countries (Jongsma et al., 2019), but comparisons have often been hampered by a lack of reliable epidemiological data (Jablensky et al., 1992). For example, while a recent report from 42 LMICs in the World Health Survey (DeVylder et al., 2018) found no consistent patterns in rural–urban differences in self-reported psychotic symptoms, it was not designed to reliably investigate psychotic disorders (Kirkbride, Keyes, & Susser, 2018). We, therefore, urgently require robust data on the incidence of psychotic disorders in settings outside of the Global North to fully characterize the epidemiology of psychotic disorders, particularly in contexts where there may be different patterns of exposure to risk factors such as poverty or rapid increases in urbanization and population density (Nations, Affairs, & Division, 2018).

To aid this effort, we analysed national register data from Chile, South America, on all people with NAPD over a 14-year period, as part of the Chilean First-Episode Schizophrenia Programme. Chile was recently categorized as a high-income country in Global South. It provides a unique setting to robustly delineate the epidemiology of NAPD for three main reasons. First, we could test whether incidence varied along well-replicated gradients in the Global North, including age, sex, multidimensional poverty and population density. We hypothesized that incidence rates would follow classic age–sex distributions and be raised in more deprived and densely populated regions. Second, our data allowed us to map changes in incidence between 2005 and 2018, during which time Chile has undergone rapid industrialization and increases in income, living standards and life expectancy. Finally, the Chilean territory spans several different latitudes, from 17° to 56° south (online Supplementary Fig. S1), which provides a unique setting to investigate the association between latitude and psychosis incidence in a single study. Various hypotheses have been proposed to link higher latitudes to greater psychosis incidence, including climatic conditions, reduced exposure to sunlight and vitamin D, genetic variation in psychosis liability, population structure and socioeconomic development (Saha, Chant, Welham, & McGrath, 2006). However, given limited direct evidence to support this hypothesis (Jongsma et al., 2018; Saha et al., 2006), we did not anticipate any association between latitude and psychosis incidence.

## Methods

### Design and settings

We used data from the Chilean First-Episode of Schizophrenia Programme (Plan de Garantías Explicitas en Salud para el primer episodio de esquizofrenia), a population-based case register covering the entire country of Chile since 2005. Chile is an ethnically homogeneous country (87% Hispanic/Caucasian), although immigration is increasing (Instituto Nacional de Estadísticas, 2017) on account of regional conflict. Since 2012, Chile has been classified as a high-income country (World Bank, n.d.), but this masks substantial inequality (NU. CEPAL, n.d.). Around 80% of the population is covered by a public health system, and all citizens (including those privately insured) have guaranteed access to treatment for NAPD under the Chilean General Guarantee in Health Law. This law took effect in 2005 and ensures rapid access to standardized treatments and financial support for 80 prevalent conditions including psychotic disorders (Letelier & Bedregal, 2006; Ministerio de Salud, 2020). We requested database access to the Public Health Sub-secretary of the Ministry of Health under the Chilean Transparency Law (registration number: AO001T0006286). We received fully anonymized data for all people with a first episode of NAPD in Chile over a 14-year period (2005–2018).

### Participants

We identified all individuals registered with a first episode of NAPD between 1 January 2005 and 29 August 2018, aged 10–64-year old, from the Chilean First-Episode of Schizophrenia Programme register. This register records all people who presented to primary, secondary or tertiary care and met clinical diagnostic criteria for the first episode of a NAPD [International Classification of Diseases, 10th revision (ICD-10), F20–29].

Participants were included regardless of where in the health system they presented (primary, secondary or tertiary care) clinical diagnoses were established by a medical doctor (primary care) or psychiatrist (secondary or tertiary care). People with a previous diagnosis of NAPD were excluded.

### Population at risk

We estimated the population at risk, aged 10–64 years old, from two Chilean national censuses in 2002 and 2017 (Instituto Nacional de Estadísticas, 2017). We used linear interpolation to estimate the population at risk for 2005–2018 for age–sex specific strata for each of the 16 regions in Chile. For 2018, we multiplied the population at risk for 2018 by 0.66 to account for person-years at risk from January to August 2018. We stratified the population by age (five-year groups; 10–14, 15–19,…, 60–64), sex, year (2005–2018) and region.

### Measures

Our primary outcome was a clinical diagnosis of ICD-10 NAPD (F20–F29). Data on age group (as above) and sex were collected at first contact for all participants. Participants were geocoded to their region at first contact. For each region, we estimated population density as the number of inhabitants per square kilometre, based on the population at-risk and total land area (Biblioteca del Congreso Nacional, 2019), excluding the sparsely populated *Antarctica* area in the Magallanes region.

We estimated regional multidimensional poverty from the Chilean Biennial National Socioeconomic Characterization Survey [*Encuesta de Caracterización Socioeconómica Nacional* (*CASEN*), 2009–2017] as the percentage of households scoring ⩾3 on 12 poverty indicators (childhood malnutrition, lack of health insurance, deficit in healthcare, school attendance, low level of education, school delay, lack of employment, lack of social security, lack of retirement, overcrowding, poor structural housing quality and deficit in basic services; online Supplementary methods) (Ministerio de Desarrollo Social, n.d.). Data for 2005–2008 were obtained from the first CASEN survey in 2009; for 2010, 2012, 2014, 2016 and 2018 we used data from the previous biennial CASEN survey. We estimated regional latitude as the midpoint for each region, ranging from 18.2° (Arica and Parinacota) to 52.3° south (Magallanes, excluding Antarctica). All regional covariates and time (in years) were treated as continuous covariates.

### Missing data

A total of 1651 of 32 358 (5.1%) participants with NAPD could not be geocoded and were excluded from indirect standardization and statistical modelling.

### Statistical analysis

We estimated crude overall treated incidence rates per 100 000 person-years and 95% confidence intervals (CIs) by socio-demographic characteristics, region and over time. Next, we used indirect standardization to compare variance in regional treated incidence. Standardized incidence ratios (SIRs) and 95% CIs were estimated based on observed and expected cases for each region, having standardized for age, sex and year by using total stratum-specific national incidence rates as our standard over the entire study period. Finally, we used random-intercepts Poisson regression to investigate multivariable variance in rates by socio-demographic, environmental factors and time, accounting for the hierarchical structure of the data set. For continuous covariates (year, multidimensional poverty, population density and latitude), we determined whether they exhibited non-linear associations with the treated incidence of NAPD by testing the fit of different fractional polynomial transformations in univariable models (online Supplementary Methods) (Royston & Sauerbrei, 2008). Age, sex and their interaction were considered *a priori* predictors of incidence in our multivariable models, with year, multidimensional poverty, population density and latitude entered in order of their univariable strength of association, assessed via Akaike's information criterion, with lower scores indicating better fit. Multivariable model improvement was assessed via the likelihood ratio test (LRT). From the final model, we estimated the linear predictor from the fixed part of the model (i.e. ignoring random effects) to plot the average marginal response (predicted natural logarithm of cases per stratum) over values of each covariate. To aid interpretation, we reported incidence rate ratios (IRRs) for multidimensional poverty and population density for *z*-standardized variables (mean = 0, standard deviation = 1), and per 10° (south) of latitude. In sensitivity analyses, we inspected the possibility that the observed non-linear association between latitude and incidence may have been driven by very low SIRs in Chile's most southerly region, Magallanes, by re-analysing the data, excluding this region. All analyses were carried out in Stata version 15 (StataCorp, 2015).

#### Ethics

Database access was approved by the Public Health Sub-secretary of Ministry of Health, through the Transparency Law of the Chilean Government (register number: AO001T0006286). The information received was anonymized and is publicly accessible.

## Results

### Participant characteristics

We identified 32 358 people with NAPD between 2005 and mid-2018, during 171.1 million person-years, corresponding to a crude incidence of 18.9 (95% CI 18.7–19.1) per 100 000 person-years. Our sample consisted of 18 573 (60.5%) men with NAPD compared to 49.6% of the population at-risk (Table 1; χ^2^ = 1440.7 on 1 degree of freedom; *p* < 0.001). Median age-at-first-contact was 24 years [interquartile range (IQR): 18–39], although this was earlier in men (23 years; IQR: 18–34 years) than women (29 years; IQR: 18–44 years; Mann–Whitney test = −20.4, *p* < 0.001). A total of 15 481 (50.4%) cases were present in primary care, 2433 (7.9%) cases in secondary care and 12 795 (41.7%) cases in tertiary care.

**Table 1.** Distribution of covariates across cases and population-at-risk

Covariate	Cases, *N* (%)	Population-at risk, *N* (%)	χ^2^ *p*-value^a^
Sex			1440.7 (1); <0.001
Men	18 573 (30.5)	84 950 222 (49.6)	
Women	12 136 (39.5)	86 148 182 (50.4)	
Age group			10212.3 (10); <0.001
10–14	2424 (7.9)	17 358 743 (10.1)	
15–19	7739 (25.2)	17 665 551 (10.3)	
20–24	5283 (17.2)	17 970 253 (10.5)	
25–29	3248 (10.6)	18 550 978 (10.8)	
30–34	2459 (8.0)	17 275 759 (10.1)	
35–39	2283 (7.4)	16 703 836 (9.8)	
40–44	2033 (6.6)	16 115 193 (9.4)	
45–49	1902 (6.2)	14 614 025 (8.5)	
50–54	1547 (5.0)	13 361 301 (8.0)	
55–59	1087 (3.5)	11 767 990 (6.9)	
60–64	704 (2.3)	9 435 776 (5.5)	
Time period			263.2 (2); <0.001
2005–2008	9779 (31.8)	48 364 485 (28.3)	
2009–2013	10910 (35.5)	62 165 517 (36.3)	
2014–2018^b^	10020 (32.6)	60 568 402 (35.4)	
Region (midpoint latitude, degrees south)^c^			1068.8 (15); <0.001
Arica (18.2°)	389 (1.3)	2 180 396 (1.3)	
Tarapaca (20.3°)	507 (1.7)	3 029 197 (1.8)	
Antofagasta (23.4°)	596 (1.9)	5 966 533 (3.5)	
Atacama (27.6°)	380 (1.2)	2 806 790 (1.6)	
Coquimbo (30.7°)	1429 (4.7)	7 027 006 (4.1)	
Valparaíso (32.8°)	3758 (12.2)	17 361 135 (10.1)	
Metropolitana de Santiago (33.4°)	12 292 (40.0)	69 627 937 (40.7)	
Libertador General Bernard O'Higgins (34.5°)	1735 (5.6)	8 808 154 (5.1)	
Maule (35.4°)	1800 (5.9)	10 107 962 (5.9)	
Bío Bío (36.6°)	1928 (6.3)	15 521 605 (9.1)	
Ñuble (37.3°)	1118 (3.6)	4 730 214 (2.8)	
La Araucanía (38.4°)	1833 (6.0)	9 345 943 (5.5)	
Los Ríos (39.7°)	985 (3.2)	3 818 596 (2.2)	
Los Lagos (42.1°)	1630 (5.3)	8 068 937 (4.7)	
Aysén (46.3°)	223 (0.7)	1 025 794 (0.6)	
Magallanes (52.3°)	106 (0.3)	1 672 206 (1.0)	
Regional population density^d^			368.6 (4); <0.001
0.9–4.6	977 (3.2)	9 308 120 (5.4)	
4.7–17.3	3683 (12.0)	18 895 848 (11.0)	
17.4–100.7	10 681 (34.8)	58 343 358 (34.1)	
100.8–432.2	6698 (21.8)	34 520 410 (30.2)	
432.3–465.6	8670 (28.2)	50 030 668 (29.2)	
Regional multidimensional poverty^e^			204.8 (3); <0.001
9.1–16.0	557 (1.8)	4 761 390 (2.8)	
16.1–23.1	12 970 (42.2)	75 766 865 (44.3)	
23.2–30.1	13 421 (43.7)	71 939 450 (42.0)	
30.2–37.2	3761 (12.2)	18 630 699 (10.9)	
Total	30 709 (100)	171 098 404 (100)	

a χ^2^ [degrees of freedom (df)]; *p* value.

b Until 28 August 2018.

c Regions are listed north to south.

d Population density is expressed as people per square kilometre.

e Regional multidimensional poverty is expressed as a percentage of households.

We observed low, non-statistically significant levels of correlation between regional multidimensional poverty, population density and latitude (online Supplementary Table S1).

### Variation in the crude incidence of NAPDs

**Fig. 1.**
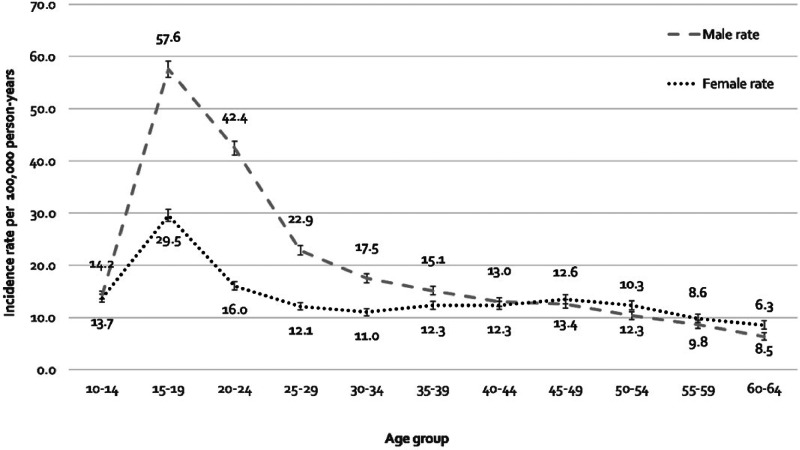
Age-distribution of the incidence of NAPDs, stratified by gender, Chile, 2005–2018.

Incidence rates by age and sex followed classic, frequently replicated shape distributions (Häfner et al., 1989; Kirkbride et al., 2006), demonstrating a strong age–sex interaction (Fig. 1; LRT χ^2^_10_: 8394.1, *p* < 0.001). Incidence rates accelerated in adolescence from low, equivocal baselines at 10–14 years old to a peak in both men (57.6 per 100 000 person-years; 95% CI 56.0–59.2) and women (29.5 per 100 000 person-years; 95% CI 28.4–30.7) between 15 and 19 years old. Rates were substantively higher for men than women from 15 to 19 years old until 40–44 years old when rates became comparable. Rates for men declined progressively after 15–19 years old, while rates for women exhibited a smaller secondary peak at 45–49 years old (13.4 per 100 000 person-years; 95% CI 12.6–14.3)

We also observed a fourfold variation in the crude incidence of NAPD across regions, from 6.3 per 100 000 person-years (95% CI 5.2–7.7) in Magallanes to 25.8 per 100 000 person-years (95% CI 24.2–27.5) in Los Rios (Table 2). Standardization for age, sex and year had a trivial impact on these estimates (Table 2).

**Table 2.** Crude and standardized incidence rates of NAPD, by region, in Chile, 2005–2018

	Crude incidence rate (95% CI)	Standardized incidence^a^ rate (95% CI)
Region (midpoint latitude, degrees south)^b^		
Arica (18.2°)	17.8 (16.1–19.7)	17.4 (15.7–19.2)
Tarapaca (20.3°)	16.7 (15.3–18.3)	16.5 (15.1–18.0)
Antofagasta (23.4°)	10.0 (9.2–10.8)	9.8 (9.1–10.7)
Atacama (27.6°)	13.5 (12.2–15.0)	13.5 (12.2–15.0)
Coquimbo (30.7°)	20.3 (19.3–21.4)	20.3 (19.3–21.4)
Valparaíso (32.8°)	21.6 (21.0–22.3)	21.5 (20.9–22.2)
Metropolitana (33.4°)	17.7 (17.3–18.0)	17.6 (17.3–18.0)
Libertador General Bernard O'Higgins (34.5°)	19.7 (18.8–20.6)	20.1 (19.2–21.1)
Maule (35.4°)	17.8 (17.0–18.6)	18.0 (17.2–18.9)
Bío Bío (36.6°)	12.4 (11.9–13.0)	12.4 (11.8–12.9)
Ñuble (37.3°)	23.6 (22.3–25.1)	24.1 (22.7–25.5)
La Araucanía (38.4°)	19.6 (18.7–20.5)	19.5 (18.6–20.4)
Los Ríos (39.7°)	25.8 (24.2–27.5)	25.6 (24.0–27.3)
Los Lagos (42.1°)	20.2 (19.2–21.2)	20.4 (19.4–21.4)
Aysén (46.3°)	21.7 (19.0–24.8)	21.9 (19.1–25.0)
Magallanes (52.3°)	6.3 (5.2–7.7)	6.4 (5.2–7.7)
Total	18.9 (18.7–19.1)	–

a Standardized for age, sex and year.

b Regions are listed north to south.

### Multilevel modelling of the incidence of NAPDs

A null multilevel model confirmed regional variation in incidence rates, indicated by a non-zero random intercepts term (*σ*^2^ = 0.11; 95% CI 0.05–0.23; *p* < 0.01; Table 3), which was not attenuated. after adjustment for individual-level age, sex and their interaction (*σ*^2^ = 0.11; 95% CI 0.06–0.23; *p* < 0.01). Univariable modelling of each regional covariate and time on the incidence of psychotic disorder suggested that population density was best-parameterized as a linear covariate (online Supplementary Table S2), but that time (in years), latitude and multidimensional poverty were best-parameterized with second-degree cubic power functions, with the shape of these parameter reported in online Supplementary Fig. S2. All regional variables were associated with the incidence of NAPD in univariable models (Table 3) with evidence that changes in incidence over time (in years) were present for women, but not men (LRT *p* value for time × sex interaction: *p* < 0.001).

**Table 3.** Univariable and multivariable random intercepts Poisson regression of NAPDs, Chile, 2005–2018

Covariate	Crude IRR (95% CI)	Fully-adjusted IRR (95% CI)	LRT *p* value^a^
Age group^b^	Sex IRR (male *v*. female)	Sex IRR (male *v*. female)	Age group × sex: 1092.5 (10); *p* < 0.001
10–14	1.04 (0.96–1.12)	0.91 (0.83, 0.99)	
15–19	1.96 (1.86–2.05)	1.70 (1.61–1.80)	
20–24	2.65 (2.49–2.81)	2.29 (2.13–2.45)	
25–29	1.89 (1.76–2.03)	1.63 (1.51–1.77)	
30–34	1.59 (1.47–1.72)	1.38 (1.26–1.50)	
35–39	1.24 (1.13–1.34)	1.07 (0.98–1.17)	
40–44	1.06 (0.97–1.15)	0.92 (0.83–1.00)	
45–49	0.94 (0.86–1.03)	0.81 (0.73–0.90)	
50–54	0.84 (0.76–0.93)	0.72 (0.64–0.80)	
55–59	0.89 (0.78–0.99)	0.76 (0.66–0.85)	
60–64	0.75 (0.63–0.86)	0.64 (0.54–0.74)	
Time			Time × sex: 96.4 (2); *p* < 0.001
Men (per year)			
First-degree cubic	0.9997 (0.9989–1.0004)	0.9997 (0.9989–1.0005)	
Second-degree cubic	1.0002 (0.9999–1.0005)	1.0001 (0.9998–1.0004)	
Women (per year)			
First-degree cubic	0.9989 (0.9984–0.9994)	0.9989 (0.9984–0.9994)	
Second-degree cubic	1.0004 (1.0002–1.0006)	1.0004 (1.0002–1.0006)	
Multidimensional poverty (*z*-standardized)			284.3 (2); <0.001
First-degree cubic	0.988 (0.985–0.992)	0.978 (0.973–0.983)	
Second-degree cubic	1.007 (1.005–1.009)	1.011 (1.009–1.014)	
Population density (*z*-standardized)	0.75 (0.66–0.85)	–^c^	0.01 (1); 0.91
Latitude (per 10° south)			10.7 (2); 0.005
First-degree cubic	1.08 (1.03–1.12)	1.08 (1.03–1.13)	
Second-degree cubic	0.96 (0.93–0.98)	0.95 (0.93–0.98)	
Random intercepts (*σ*^2^)	0.11 (0.06–0.23)	0.07 (0.03–0.14)	

a From fully-adjusted model. LRT χ^2^ [degrees of freedom (df)]; *p* value.

b For IRR for men and women by age group *v.* 10–14-year-old (reference), see online Supplementary Table S3.

c Not retained in the final model (IRR: 0.98; 95% CI 0.88–1.10).

**Fig. 2.**
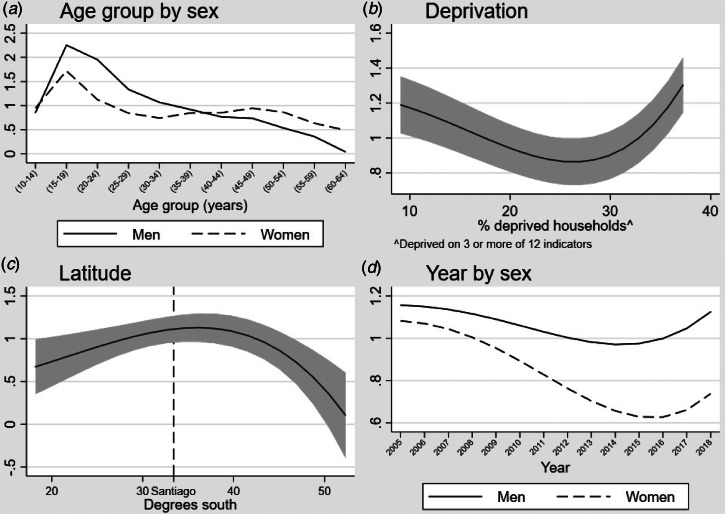
Non-linear relationships between selected risk factors and predicted cases of NAPDs in Chile, 2005–2018.

Our best-fitting multivariable model of NAPD included age, sex, their interaction, multidimensional poverty, latitude and time (in years) and its interaction with sex (Table 3). Thus, in our fully-adjusted model, we observed strong age–sex trends in predicted incidence cases (Fig. 2*a*), with statistically significant excess IRRs in men compared with women between 15 and 34 years old and in women compared with men at 10–14 years old and after 45 years old (Table 3). Regional multidimensional poverty exhibited a strong non-linear u-shaped association with incidence (Fig. 2*b*), such that rates were highest in the poorest and most affluent regions of Chile, although this pattern was most pronounced in Chile's poorest regions. Increased latitude (i.e. greater distance from the equator) was initially associated with an increased incidence of NAPD, which continued beyond Chile's capital and most densely populated city, Santiago (Fig. 2*c*) until ~36° south, before declining sharply at latitudes closer to Magallanes. As in crude models, incidence rates declined over time for women, but not men (LRT; *p* < 0.001), with both rates exhibiting a non-linear association, demonstrating an initial decline followed by rising rates after 2016 (Fig. 2*d*). Regional population density did not improve model fit and was not associated with NAPD incidence after adjustment for all other covariates (IRR 0.98; 95% CI 0.88–1.10). Our final model suggested some residual variance in incidence rates at the regional level remained present (*σ*^2^ = 0.07; 95% CI 0.03–0.14; *p* < 0.01).

### Sensitivity analyses

To exclude the possibility that the observed non-linear association between latitude and incidence may have been driven by very low age–sex standardized incidence rates in Chile's most southerly region, Magallanes (Table 2), we re-analysed the data excluding this region. In these analyses, we obtained identical shape parameters between psychosis incidence and time, population density and multidimensional poverty, but latitude was best-parameterized as a second-degree inverse polynomial function of the form *x*^(−1,−1)^. This indicated a broadly linear univariable relationship with the incidence of NAPD (see online Supplementary Fig. S3). However, after excluding Magallanes, we found no evidence in multivariable modelling that latitude (LRT; *p* = 0.20) was associated with NAPD incidence, after adjustment for time, age group and their interactions with sex and regional multidimensional poverty (online Supplementary Table S4).

## Discussion

### Principal findings

We have precisely characterized the incidence of NAPD at a national level outside of the Global North for the first time, by delineating rates for 10–64 years old in Chile over a 14-year period. Incidence rates revealed typical variation by age and sex as identified in previously published findings in other contexts. (Häfner et al., 1989; Kirkbride et al., 2006) We also provided further evidence that the incidence of NAPD follows a non-linear association with multidimensional poverty. This was independent of pronounced non-linear variation by latitude, where rates initially increased with distance (south) from the equator, before decreasing below ~36° south. Rates decreased over time for women, but not men, following non-linear relationships. After adjustment for these factors, there was no association between the incidence of NAPD and regional population density.

### Comparison with the previous literature

Our overall estimate of the crude incidence of NAPD in Chile (18.9 per 100 000 person-years; 95% CI 18.7–19.1) was broadly similar to the wider literature, both globally and within the region. For example, the most recent international meta-analysis on this topic estimated a pooled incidence of NAPD of 18.7 (95% CI 17.8–23.6) per 100 000 person-years across studies conducted between 2002 and 2017 (Jongsma et al., 2019), a similar time period to the present study. The treated rate of NAPD in Chile was marginally higher than the overall rate reported in the multinational EU-GEI study of 16.9 per 100 000 person-years (95% CI 16.2–17.6), and higher than comparable data from Ribeirao Preto in Brazil at 14.8 (95% CI 13.4–16.3) (Jongsma et al., 2018).

We observed a fourfold variation in age–sex standardized incidence rates across 17 broad regions in Chile. In contrast to the international comparisons above, which present remarkably similar estimates of the overall incidence of NAPD, subnational variation in rates is frequently reported between settings (Jongsma et al., 2018; March et al., 2008). For example, the pooled rate estimated from the recent aforementioned meta-analysis was accompanied by substantial heterogeneity across studies (*I*^2^ = 99.6%), indicative of setting-level variation. Similarly, across 17 settings in the six-country EU-GEI study, there was eightfold variation in age–sex–minority standardized rates of NAPD. One potential explanation for the greater variability observed in the EU-GEI study than in our nationwide study is that we were able to conduct our study across a single healthcare system, minimising variance due to hard-to-capture differences in healthcare systems, and referral and case ascertainment patterns inherent in any international study.

The higher rates of NAPD we observed in young men between 15 and 34 years old are also frequently reported in the literature (Kirkbride et al., 2012; Okkels, Vernal, Jensen, McGrath, & Nielsen, 2013). As in previous studies, rates for men and women declined dramatically from peak incidence rates at 15–19 years, such that by 35 years old, adjusted rates for men and women were equivocal (online Supplementary Table S3). The median age at presentation in our study (24 years old) was younger than reported in other samples, such as the EU-GEI study (30.5 years old; Jongsma et al., 2018), but this difference may be explained by restriction of our sample to non-affective psychoses, which may have an earlier median age at first presentation than affective psychoses (Kirkbride et al., 2017).

To our knowledge, only one previous study has estimated the national incidence of psychotic disorders before 15 years old. That study, based on a narrower definition of schizophrenia, reported lower incidence rates before 15 years old between 1971 and 2010 than reported here for all NAPD. Our data suggest that treated incidence rates in early adolescence in Chile were non-negligible and comparable to those for women and men aged ⩾25 and ⩾35 years, respectively. As previously reported in Denmark, we found slightly higher incidence rates before 15 years old in women than men, consistent with sex ratios observed for psychotic experiences reported in the general population at this age (Zammit et al., 2014). Given our large sample size, we also had high precision to extend previous findings on rates at older ages, providing the strongest evidence to date that rates continue to decline more rapidly for men than women after 45 years old, such that rates for men were almost half that of women after 60 years old. A recent study from Sweden on the incidence of NAPD occurring after 60 years old suggests that incidence rates may begin to increase after this age (Stafford, Howard, & Kirkbride, 2018), and particularly for women beyond 75 years old. Together, these findings warrant increased attention to the burden of NAPD in older groups and women.

In Chile, there is no community-based early intervention in psychosis services. This could explain our finding that half (50.4%) of the cases presented in primary care and 41.7% in tertiary care. These results demonstrate the relevance of incorporating primary care in accurate and timely detection and diagnosis of NAPD, underscoring findings from the Global North (Sullivan et al., 2018).

Our study captures the broad spectrum of first-episode NAPD incidence rates, providing essential information for the Chilean Health System to allocate adequate resources for the clinical follow-up of this patient population and to strengthen and improve the current Chilean First-Episode Schizophrenia Programme.

Our paper adds further evidence to support previous observations that the association between multidimensional poverty and the incidence of NAPD is non-linear. Such patterns were first reported in the UK (Croudace, Kayne, Jones, & Harrison, 2000), and have been recently replicated (Kirkbride et al., 2017; O'Donoghue et al., 2016). It remains unclear whether these findings reflect aetiological differences in risk (Sariaslan et al., 2015), On the one hand, the highest rates in Chile's poorest regions is consistent with a role for several factors, including infections, obstetric complications or exposure to social disadvantage. However, slightly higher rates observed in more affluent regions mean we cannot exclude the possibility that multidimensional poverty also affects access to healthcare (e.g. those living in wealthier regions are more likely to access healthcare) to contribute to the patterns of treated incidence we observed. In this regard, our results could inform Chilean public mental health by revealing spatial patterns in potential future need for early intervention care for psychosis, as has been demonstrated using robust psychosis epidemiology in other contexts (Kirkbride et al., 2013).

We also observed a non-linear association between the incidence of NAPD and distance from the equator in Chile. This did not follow a simple linear increase in risk with distance from the equator, as suggested in the literature (Jongsma et al., 2018; Saha et al., 2006), perhaps arguing against any direct bio-meteorological explanations. We cannot exclude the possibility that this association was attributable to differential case ascertainment between regions, particularly with the sharp decline in incidence at higher latitudes in some of Chile's remotest southern regions (i.e. Magallanes). The Magallanes region is the second least populated region in Chile (after neighbouring Aysén to the north) and has the lowest levels of poverty of all Chilean regions given a strong economy based on oil, sheep farming and tourism. The region is well-served by public health facilities and 80% of the population live in the regional capital, Punta Arenas, making case ascertainment an unlikely explanation of the low rates here. Possible explanations for these low rates may be the socioeconomic affluence of the region or its low levels of urbanicity. Nonetheless, in our study, the regional population density was not associated with the incidence of NAPD. Population density has been frequently associated with the NAPD incidence in studies predominantly from Northern Europe and Canada (March et al., 2008). More recent reports have suggested such gradients may not hold in all settings (Del-Ben et al., 2019; Jongsma et al., 2018), partially supported by evidence on the prevalence of subclinical psychotic experiences (DeVylder et al., 2018). To our knowledge, ours is the first nationwide study to investigate this issue outside of the Global North.

Our finding that the incidence of NAPD decreased over time – most depreciable amongst women – has not been observed elsewhere. This change coincided with substantial economic growth over the study period, as Chile transitioned from a middle-income to a high-income country. The particularly strong decline in women remains unexplained. A potential explanation could be a diagnostic bias if women were more likely to be diagnosed with affective psychotic disorders over time. Unfortunately, the current database was restricted to non-affective psychoses, but this underscores the importance of providing early intervention services for the spectrum of all psychotic disorders. This would support timely, gender-balanced clinical intervention and routine data collection to fully investigate such issues.

### Strengths and limitations

Our findings should be interpreted alongside the strengths and limitations of our study. We used a single, large comprehensive national case register covering the entire health system. This allowed us to estimate the incidence of NAPD with a high degree of precision. We also included a wide age-range, starting at age 10, which is uncommon in incidence studies. Nonetheless, some limitations of our study design need to be acknowledged. Our rate estimates should be interpreted as treated incidence rates from those present in the health system in Chile, although the detection of cases who never present to services is not unique to the present study and healthcare access in Chile is good (Barber et al., 2017). We also had to rely on clinical diagnoses at first presentation, without recourse to any standardized instrument. To minimize diagnostic uncertainty, we purposively used the broad diagnostic category of all NAPDs. Clinical diagnoses have been demonstrated to be reliable in other settings (Dalman, Broms, Cullberg, & Alleback, 2002) and our estimates are in line with the existing literature, providing some face validity to our findings. Nonetheless, it is essential that future studies in Chile assess the validity and reliability of these register-based diagnoses. Our geographical analyses of variance in incidence rates were restricted to broad regions, which may have obscured elucidation of smaller area effects on the incidence of NAPD. Finally, due to the available data, we could not investigate the role of migrant status or ethnicity.

## Conclusions

We have characterized variance in the incidence of NAPD in a nationwide register study in the Global South for the first time. Our replication of well-established variation in rates by age and sex underscore the differential risks exhibited for men and women over the life course and should inform public mental health policymakers about the need for timely access to early intervention for psychosis. As elsewhere, our results suggest NAPD rates are elevated in the very poorest regions and provide a target for effective resource allocation to address such disparities. Finally, we found no evidence of an association with population density in Chile, suggesting this may be a context-dependent risk factor for NAPD.

## Supplementary material

For supplementary material accompanying this paper visit https://doi.org/10.1017/S0033291720002664. click here to view supplementary material
