# Supplementary material for: The incidence of non-affective psychotic disorders in Chile between 2005 and 2018: results from a national register of over 30 000 cases
Source: Psychol Med. 2020 Aug 6;52(5):914–23. doi: 10.1017/S0033291720002664 (PMC9005445; doi:10.1017/S0033291720002664)
Supplement: Supplementary file 1 [file S0033291720002664sup001.docx]

**Supplemental Materials**

**Supplemental Figure 1: Map of Chile by regional subdivisions**


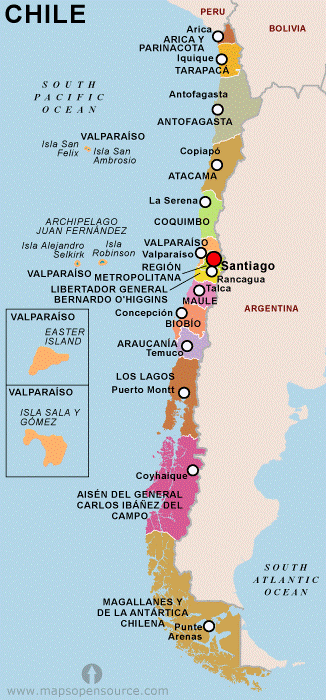


**Supplemental Methods**

*Multidimensional poverty*

Multidimensional poverty has been used by the Chilean government since 2009 as a measure of poverty and is estimated using The National Socioeconomic Characterization Survey (CASEN, Encuesta de Caracterización Socioeconómica Nacional). This survey uses household as analysis unit and assesses four broad dimensions - health, education, work/social security and housing. Each dimension is subdivided into three poverty indicators.

- *Health* is divided in i) malnutrition (if there is a child with obesity or undernutrition); ii) lack of health protection (if one of household´s member does not have health insurance); and iii) deficit in healthcare (if one of household´s member did not receive medical care in the last three months due to external reasons, such as severe delay on medical attention).
- *Education* is divided in i) school attendance (if there is household´s member between four and eighteen years is not attending to school); ii) low level of education (if one of household´s member have not completed at least twelve years of minimal formal education); and iii) school delay (if any of household´s member with twenty-one years old or less is attending to school and is delayed two or more years).
- The *work and social security* dimension is divided into i) lack of employment (if any of non-student household´s member, older than eighteen years old, is unemployed and is looking for a job); ii) lack of social security (if any of worker´s household member older than eighteen years does not have a social security income); and iii) problems with retirement (if any of retired household´s member does not: receive a pension).
- The *housing* dimension is divided in i) overcrowded (the number of people per each room is more than 2,5); ii) poor structural house quality, and iii) deficit in basics services, such as poor access to clean drinking water.

Every poverty indicator has the same weighting, and a household is considered to live in multidimensional poverty if criteria for three or more of these twelve indicators are met.

*Fractional polynomial transformations.*

For continuous covariates (year, deprivation, population density and latitude) we determined whether they exhibited non-linear associations with the incidence of non-affective psychoses by testing the fit of different fractional polynomial transformations in univariable models. We considered up to second degree fractional polynomial functions over a range of powers {-0.5,0,0.5,1,2,3} permitting a flexible range of curve shapes to be tested, as recommended by Royston and Sauerbrei.^1^ Two-dimensional fractional polynomials take the form:

$$x^{(p_{1},p_{2})'}\beta= \beta_{0}+\beta_{1}x^{(p_{1})}+\beta_{2}x^{(p_{2})}\ln\left( x \right)$$

and in the special case where:

$$p= 0$$

then:

$$x^{(0)}= \ln\left( x \right)$$

The fractional polynomial transformation of each variable which provided best fit to the incidence data in a univariable regression, assessed via likelihood ratio test [LRT], was used during multivariable modelling.

**Supplemental Results**

**Supplemental Figure 2: Fractional polynomial parameterizations for regional covariates and time (in years) from univariable models^1^**


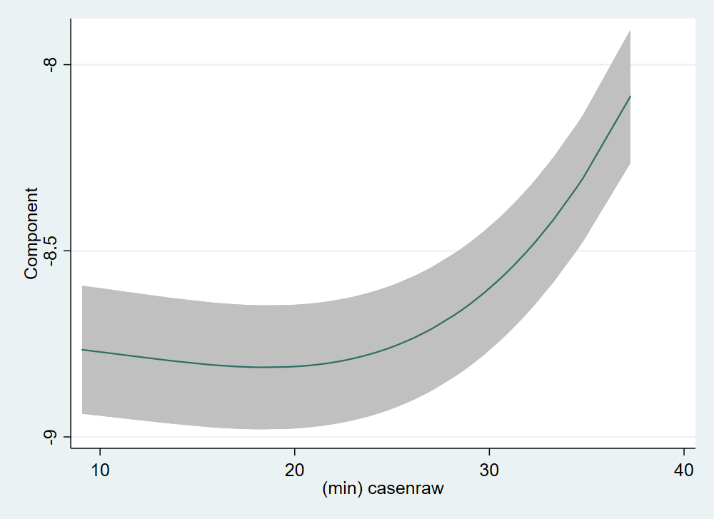

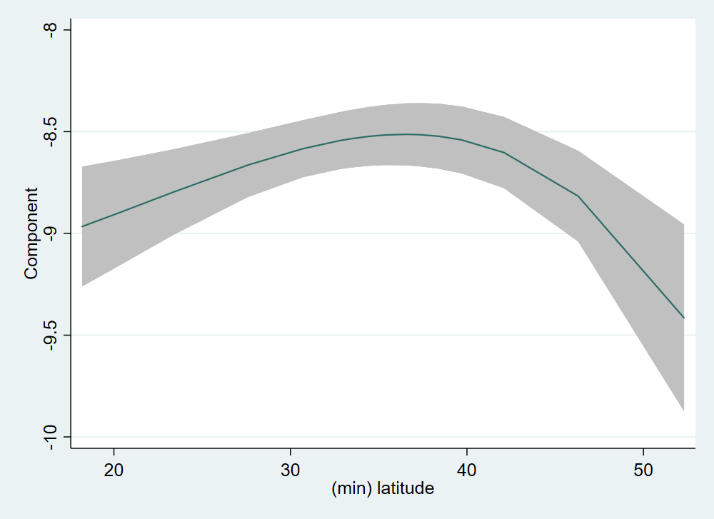

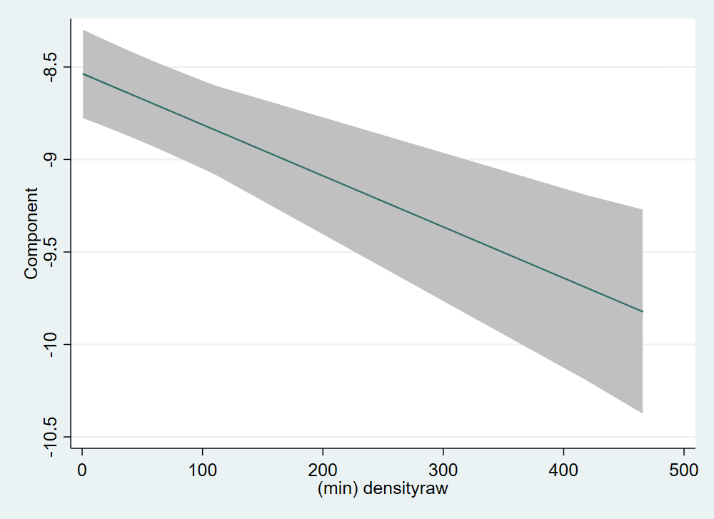

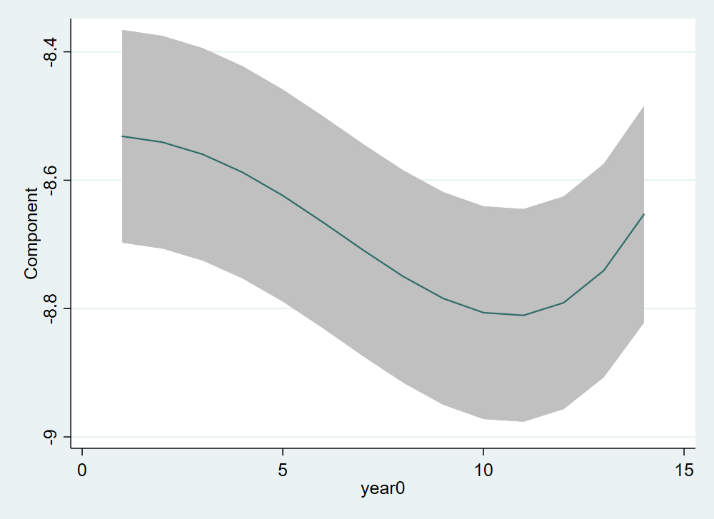


**(a) Multidimensional poverty**

**(b) Latitude**

**(c) Time (years)**

**(d) Population density**

^1^Multidimensional poverty, latitude and time (years) were all best-parameterized with a second-degree cubic function (see Supplemental Table 2); population density was best modelled as a linear term.

**Supplemental Figure 3: Re-estimated fractional polynomial parameterization for univariable association between latitude and incidence of non-affective psychoses in Chile, excluding Magallanes region in sensitivity analyses^1^**

**
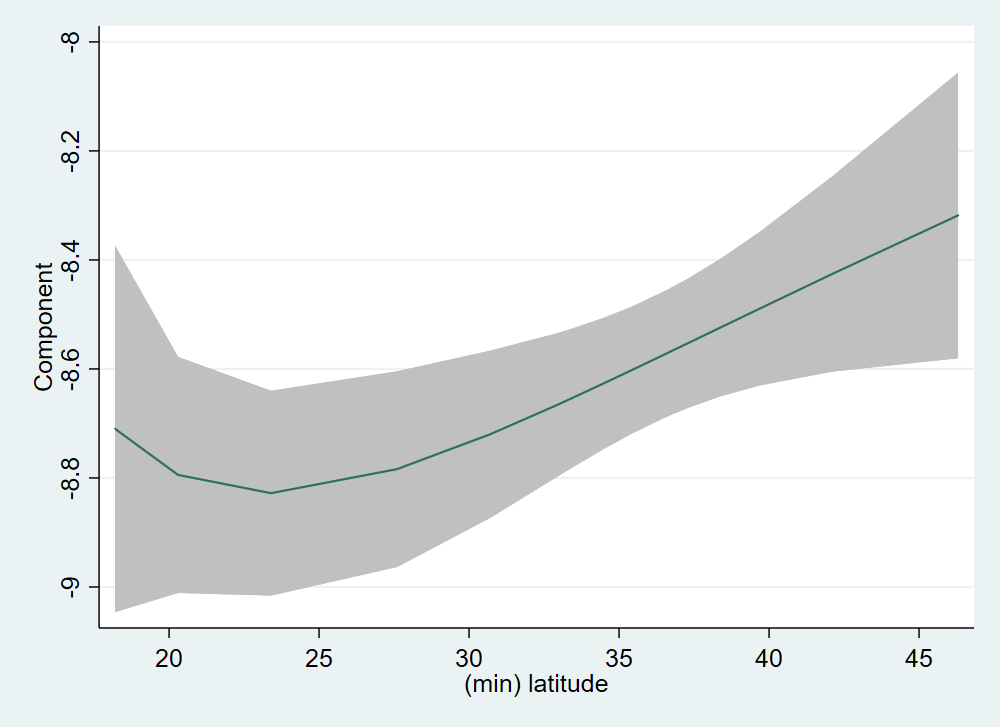
**

^1^After excluding the Magallanes region, the univariable association between latitude and the incidence of non-affective psychoses in Chile, 2005-2018, was best-parameterized with a $x^{(-1,-1)'}\beta$ fractional polynomial term

**Supplemental Table 1: Correlation between regional variables in 2018, Chile (N=17)**

| **Covariate** | **Median (IQR)** | **Range** | **Correlation^1^** | | |
| --- | --- | --- | --- | --- | --- |
|  |  |  | Population density | Deprivation | Latitude |
| Population density (ppkm^2^) | 19.9 (6.4-46.5) | 1.0-465.6 | - |  |  |
| Multidimensional poverty^2^ (%) | 19.9 (17.1-21.5) | 10.7-21.5 | -0.13 | - |  |
| Latitude (degrees south) | 35.0 (29.2-39.1) | 18.2-52.3 | -0.02 | -0.36 | - |

IQR: Interquartile range; ppkm^2^: people per square kilometre

^1^No correlation met statistical significance at p<0.10

^2^Proportion of households in each region living in multidimensional poverty

**Supplemental Table 2: Best-fitting fractional polynomial functions for selected covariates in univariable models of the incidence of non-affective psychoses, Chile, 2005-2018**

| **Covariate** | **Best-fitting term (x^p1,p2^)** | **Improvement over linear (i.e. x^1^)** |
| --- | --- | --- |
| Population density | Linear (x^1^) | - |
| Multidimensional poverty | (x^3,3^) | <0.001 |
| Latitude | (x^3,3^) | <0.001 |
| Year | (x^3,3^) | <0.001 |

| **Covariate** | **Men**  **IRR (95%CI)** | **Women**  **IRR (95%CI)** |
| --- | --- | --- |
| Age group |  |  |
| 10-14 | 0.25 (0.23, 0.27) | 0.46 (0.43, 0.49) |
| 15-19 | Ref | Ref |
| 20-24 | 0.74 (0.71, 0.77) | 0.55 (0.52, 0.59) |
| 25-29 | 0.40 (0.38, 0.42) | 0.42 (0.39, 0.45) |
| 30-34 | 0.31 (0.29, 0.32) | 0.38 (0.35, 0.41) |
| 35-39 | 0.26 (0.25, 0.28) | 0.42 (0.39, 0.45) |
| 40-44 | 0.23 (0.21, 0.24) | 0.42 (0.39, 0.45) |
| 45-49 | 0.22 (0.20, 0.24) | 0.46 (0.43, 0.50) |
| 50-54 | 0.18 (0.17, 0.19) | 0.42 (0.39, 0.46) |
| 55-59 | 0.15 (0.14, 0.16) | 0.34 (0.31, 0.37) |
| 60-64 | 0.11 (0.10, 0.12) | 0.29 (0.26, 0.32) |

**Supplemental Table 3: Age-specific incidence rate ratios for men and women from fully-adjusted model, Chile, 2005-2018^1^**

^1^Alternative parameterization of age-sex interaction term from fully-adjusted model reported in Table 3, additionally adjusted for regional multidimensional poverty, latitude and time x sex.

**Supplemental Table 4: Sensitivity analyses excluding the Magallanes region from multivariable modelling**

| **Covariate** | **Full sample^1^**  **IRR (95%CI)** | **Exc. Magellanes^2^**  **IRR (95%CI)** | **LRT p-value (exc. Magallanes)^3^** |
| --- | --- | --- | --- |
| Age group | *Sex IRR (M vs. F)* | *Sex IRR (M vs. F)* | Age x sex: |
| 10-14 | 0.91 (0.82, 0.99) | 0.91 (0.83, 0.99) | 1084.4 (10); <0.001 |
| 15-19 | 1.53 (1.48, 1.59) | 1.70 (1.60, 1.80) |  |
| 20-24 | 1.83 (1.76, 1.90) | 2.29 (2.13, 2.44) |  |
| 25-29 | 1.49 (1.41, 1.57) | 1.63 (1.50, 1.76) |  |
| 30-34 | 1.32 (1.24, 1.41) | 1.38 (1.26, 1.50) |  |
| 35-39 | 1.07 (0.98, 1.16) | 1.07 (0.98, 1.17) |  |
| 40-44 | 0.91 (0.82, 1.01) | 0.91 (0.83, 1.00) |  |
| 45-49 | 0.79 (0.70, 0.89) | 0.81 (0.73, 0.89) |  |
| 50-54 | 0.67 (0.57, 0.78) | 0.72 (0.64, 0.80) |  |
| 55-59 | 0.72 (0.60, 0.85) | 0.76 (0.66, 0.85) |  |
| 60-64 | 0.55 (0.40, 0.71) | 0.64 (0.54, 0.74) |  |
| Time |  |  | Time x year: |
| Men (per year) |  |  | 97.1 (2); <0.001 |
| First-degree cubic | 0.9997 (0.9989, 1.0005) | 0.9998 (0.9990, 1.0006) |  |
| Second-degree cubic | 1.0001 (0.9998, 1.0004) | 1.0001 (0.9998, 1.0004) |  |
| Women (per year) |  |  |  |
| First-degree cubic | 0.9989 (0.9984, 0.9994) | 0.9989 (0.9984, 0.9993) |  |
| Second-degree cubic | 1.0004 (1.0002, 1.0006) | 1.0004 (1.0002, 1.0006) |  |
| Multidimensional poverty (z-standardised) |  |  | 283.3 (2); <0.001 |
| First-degree cubic | 0.978 (0.973, 0.983) | 0.980 (0.976, 0.984) |  |
| Second-degree cubic | 1.011 (1.009, 1.014) | 1.010 (1.008, 1.012) |  |
| Population density (z-standardised) | -^4^ | -^4^ | 0.14 (1); 0.70 |
| Latitude (per 10 degrees south) |  |  | 3.23 (2); 0.20 |
| First-degree^3^ | 1.08 (1.03, 1.13) | -^5^ |  |
| Second-degree^3^ | 0.95 (0.93, 0.98) | -^5^ |  |
| Random intercepts (σ^2^) | 0.07 (0.03, 0.14) | 0.07 (0.03, 0.14) |  |

^1^As reported in Table 3. Included here for comparison only.

^2^Data re-analysed excluding Chile’s most southerly region, *Magallanes*, with low incidence rates of non-affective psychoses, to inspect role of non-linear latitude on results

^3^LRT χ^2^ (degrees of freedom [df]); p-value

^4^Not retained in the final model for the full sample (IRR: 0.98; 95% CI: 0.88, 1.10) or the sample excluding *Magallanes* (IRR: 1.02; 95% CI: 0.92, 1.14)

^5^Cubic in the full sample model, better parameterized as a two-degree inverse power function of the form $x^{(-1,-1)'}\beta$ (see Supplemental Figure 2), but not statistically-significant in final model at p<0.05. IRR estimates for the first- and second-degree inverse power of latitude (per 10 degrees) were 1.02 (95% CI: 0.96, 1.09) and 0.99 (95% CI: 0.95, 1.03), respectively.

**Supplemental Bibliography**

^1^ Royston P, Sauerbrei W. Multivariable Model-Building: A Pragmatic Approach to Regression Analysis Based on Fractional Polynomials for Modelling Continuous Variables. Chichester, West Sussex: Wiley; 2008.
